# Associations Between Social Isolation, Education, and Rurality and Cognitive Health in the LASI Study

**DOI:** 10.1093/geroni/igaf122.4291

**Published:** 2025-12-31

**Authors:** Raaga Likhitha Musunuri, Kavya Vytla, Kajal Dhansingh Bhauryal, Aarushi Vatsyayan, Amit Kumar, Nasim Ferdows

**Affiliations:** Northeastern University, Boston, Massachusetts, United States; Northeastern University, Boston, Massachusetts, United States; Northeastern University, Boston, Massachusetts, United States; Northeastern University, Boston, Massachusetts, United States; University of Utah, Salt Lake City, Utah, United States; Northeastern University, Boston, Massachusetts, United States

## Abstract

Social isolation is increasingly recognized as a risk factor for cognitive decline, yet its measurement and implications remain underexamined in low- and middle-income countries. This study examines various indicators of the multidimensional Social Isolation Index (SII) and its association with cognitive functioning among older adults in India. We used nationally representative data from the Longitudinal Aging Study in India (LASI) Wave 1 (2017–2019), focusing on individuals aged ≥60 years (N = 31,300) across 34 states and union territories. The SII combined six indicators (marital status, household structure, child contact, social and religious participation, and loneliness) into a weighted index (0-1). Cognitive function was measured using the composite cognitive index score (0-43). Linear regression models were used to assess the association between SII and cognition after sequential adjustment for socio-demographic, health, and behavioral covariates. Moderation was tested through interaction terms with rurality and education. A higher SII score was associated with lower cognitive functioning, with a 1.04-point difference between the most and least isolated individuals (95% CI: −1.13, −0.96). This association remained robust after full adjustment (−0.67, 95% CI: −0.74, −0.59). No rural-urban differences were observed. Educational attainment moderated the association, particularly in urban settings, where the association was weaker among those with higher education. SSI is consistently associated with lower cognitive functioning in later life, with education appearing to modify this association, particularly in urban settings. Understanding the effects of both objective and subjective dimensions of isolation is crucial for developing strategies to support healthier aging and improve cognitive health.

